# Genetic relationships and genome selection signatures between soybean cultivars from Brazil and United States after decades of breeding

**DOI:** 10.1038/s41598-022-15022-y

**Published:** 2022-06-23

**Authors:** João Vitor Maldonado dos Santos, Gustavo Cesar Sant’Ana, Philip Traldi Wysmierski, Matheus Henrique Todeschini, Alexandre Garcia, Anderson Rotter Meda

**Affiliations:** Tropical Melhoramento & Genética (TMG), 87 Celso Garcia Road, Cambe, PR Brazil

**Keywords:** Genetics, Agricultural genetics, Genetic markers, Genomics, Plant breeding, Plant genetics

## Abstract

Soybean is one of the most important crops worldwide. Brazil and the United States (US) are the world’s two biggest producers of this legume. The increase of publicly available DNA sequencing data as well as high-density genotyping data of multiple soybean germplasms has made it possible to understand the genetic relationships and identify genomics regions that underwent selection pressure during soy domestication and breeding. In this study, we analyzed the genetic relationships between Brazilian (N = 235) and US soybean cultivars (N = 675) released in different decades and screened for genomic signatures between Brazilian and US cultivars. The population structure analysis demonstrated that the Brazilian germplasm has a narrower genetic base than the US germplasm. The US cultivars were grouped according to maturity groups, while Brazilian cultivars were separated according to decade of release. We found 73 SNPs that differentiate Brazilian and US soybean germplasm. Maturity-associated SNPs showed high allelic frequency differences between Brazilian and US accessions. Other important loci were identified separating cultivars released before and after 1996 in Brazil. Our data showed important genomic regions under selection during decades of soybean breeding in Brazil and the US that should be targeted to adapt lines from different origins in these countries.

## Introduction

Soybean [*Glycine max* (L.) Merrill] is one of the most important crops worldwide. It contributes to oil production as well as human and animal diets^1^. Brazil and the United States (US) were responsible for more than 60% of the world soybean production in the 2020–2021 growing season. Brazil had a soybean production of 138.2 million tons harvested from 39.2 million hectares of cultivated area, and the US was responsible for 120.7 million tons harvested from 34.9 million hectares of cultivated area^2,3^. The Brazilian soybean-breeding programs have a relatively brief history, with US germplasm introduction starting in the 1940s and becoming economically important after the 1970s with the discovery of genes related to the long juvenile period trait^4^. The US soybean-breeding programs have an older history than the Brazilian breeding programs. Soybeans were introduced in the US at the beginning of the 1900s, but only became important as an oilseed crop after World War II^5^.

Despite advances of the soybean-breeding programs in germplasm improvement, some important factors limit crop production. One of the biggest challenges is the narrow genetic base observed in soybean germplasm. According to a pedigree analysis, the US genetic base was basically generated by 35 soybean genotypes^6^. In another study, a similar analysis found that Roanoke, S-100, CNS, and Tokyo contributed to 55.3% of the Brazilian genetic base. Furthermore, Brazilian and US germplasms share six ancestors: CNS, S-100, Roanoke, Tokyo, PI 54,610, and PI 548318^7^. A genomic study with 28 Brazilian soybean accessions suggested that the genetic base remains narrow despite some diversified genomic regions^8^.

Next-generation sequencing methods have become an important tool to increase soybean genome knowledge^9,10^. The first reference genome for soybean was assembled based on the ‘Williams 82’ cultivar, with 46,430 protein-coding genes distributed on 20 chromosomes with approximately 978-megabases (Mb) in total size^11^. Recently, two other reference genomes were generated in soybean: the Chinese accession ‘Zhonghuang 13’ (with a reference genome of 1,025 Mb total size and 52,051 protein-coding genes)^12^ and the cultivar ‘Lee’ (with approximately 1,015-Mb of total size)^13^. The existence of reference genomes in soybean facilitated the publication of a large number of studies associated with diversity and population analysis, allelic variation discovery and genome-wide association studies (GWAS)^9^.

In this context, the objectives of this study were to analyze genetic kinship relationships between Brazilian and US soybean cultivars from different maturity groups (MG) and release dates as well as to identify genome selection signatures between and within Brazilian and US cultivars.

## Results

### Different structures were detected between the Brazilian and US genetic bases

**Figure 1:**
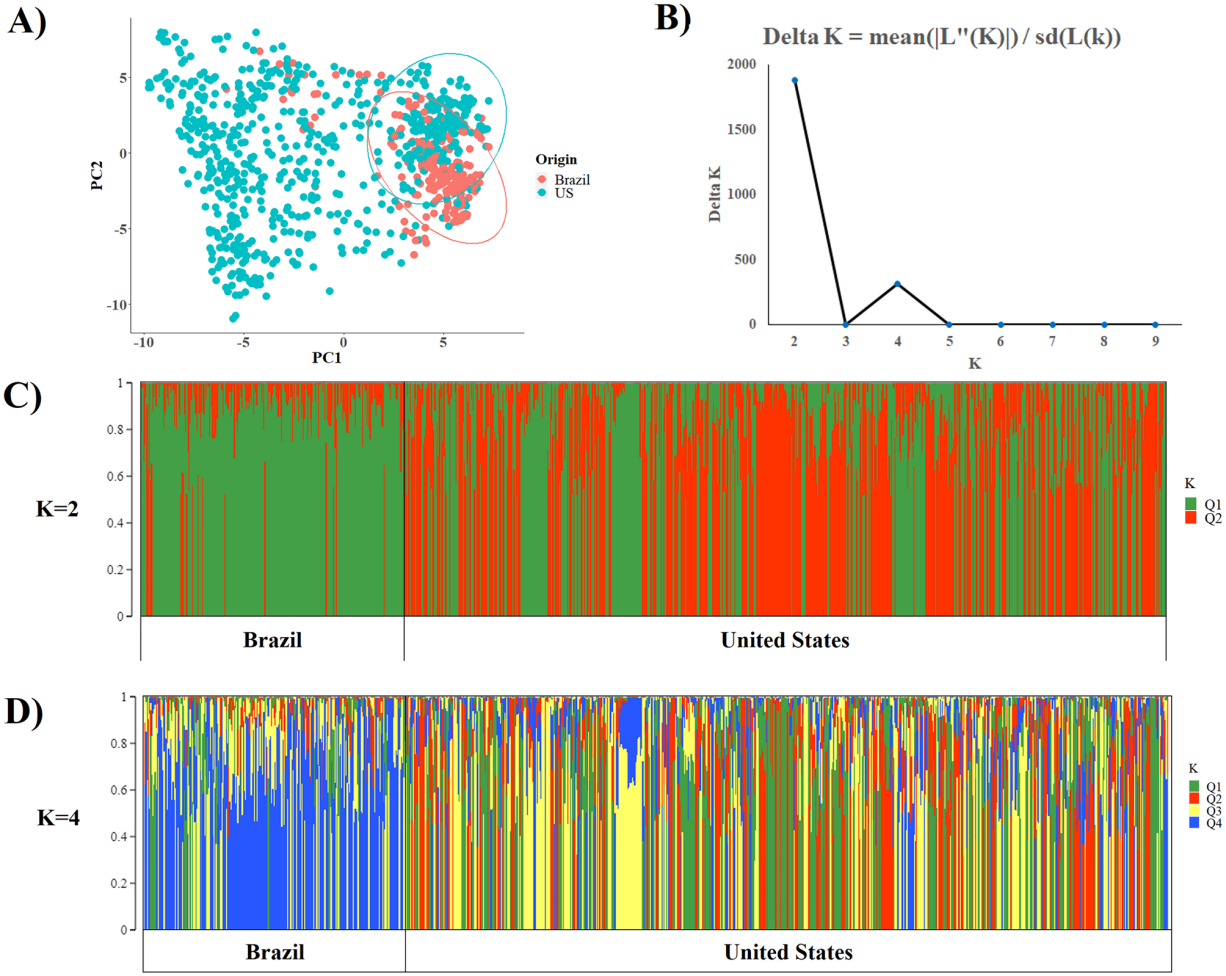
Population structure analysis between Brazilian and US germplasms. (**A**) Principal component analysis of Brazilian and US soybean cultivars based on SNPs markers; **(B**) Delta K as a function of the number of groups (K); (**C**) assignment coefficients of individual cultivars (bar plots) considering K = 2; and (**D**) considering K = 4.

Principal component analysis (PCA) revealed that most Brazilian cultivars (red circle) were grouped with a subgroup of US cultivars (green circle). Most of them belonged to MG VI, VII, VIII and IX (Fig. 1A). Based on the Evanno criterion (Fig. 1B), the structure results based on four groups (K = 4) showed a high ΔK value (312.35), but the upper-most level of the structure was in two groups (K = 2; ΔK = 1885.43).

Considering K = 2 (Fig. 1C), the Brazilian cultivars jointly presented an assignment to the Q1 group (green) equal to 86.7% which was much higher than that observed for the US cultivars (43.9%). Considering K = 4 (Fig. 1D), the Brazilian cultivars jointly presented an assignment to the Q2 group (red) of only 4.7% while the US cultivars jointly presented an assignment to the Q2 group of 27.4%. The Q1 group (green) has a lower assignment in Brazilian cultivars than US accessions (11.1%, and 30.1%, respectively). These results demonstrate that the set of Brazilian cultivars has a narrower genetic base compared to US cultivars.

### Large genetic divergences between the Brazilian and US soybean germplasms were observed according to their maturity groups

When we compared the cultivars between maturity groups, we observed a clear differentiation between early and late groups. The highest genetic distances (0.4158) observed were between MG 00–0 and MG VIII-IX cultivars (Supplementary Table S1).

To examine the influence of maturity groups on population structure, we analyzed the average assignment coefficients (K = 4) of Brazilian and US cultivars for each maturity group (Supplementary Figure S1). Brazilian cultivars from maturity group V presented Q1, Q2, Q3, and Q4 equal to 30.4%, 1.9%, 32.1, and 32.0%, respectively; US cultivars from this same maturity group (V) presented means of Q1, Q2, Q3, and Q4 equal to 9.2%, 8.2%, 65.1%, and 17.6%, respectively. This result indicates that, although belonging to the same maturity group, the Brazilian group V cultivars present considerably different allelic frequencies than the US cultivar group V cultivars, especially for Q3 and Q4. US cultivars belonging to earlier maturity groups (00, 0, I, and II) had significantly higher mean assignment coefficient to Q2 group (red) compared to other later maturity groups (V = 8.2%, VI = 8.1%, VIII = 5.0%, and IX = 13.6%). In the case of Brazilian cultivars, the average assignment coefficients for Q2 were much lower (V = 1.9%, VI = 4.2%, VII = 5.6%, VIII = 4.9% and IX = 4.9%). These results demonstrate an important allelic pool that distinguishes early to late genetic materials present in Q2.

**Figure 2:**
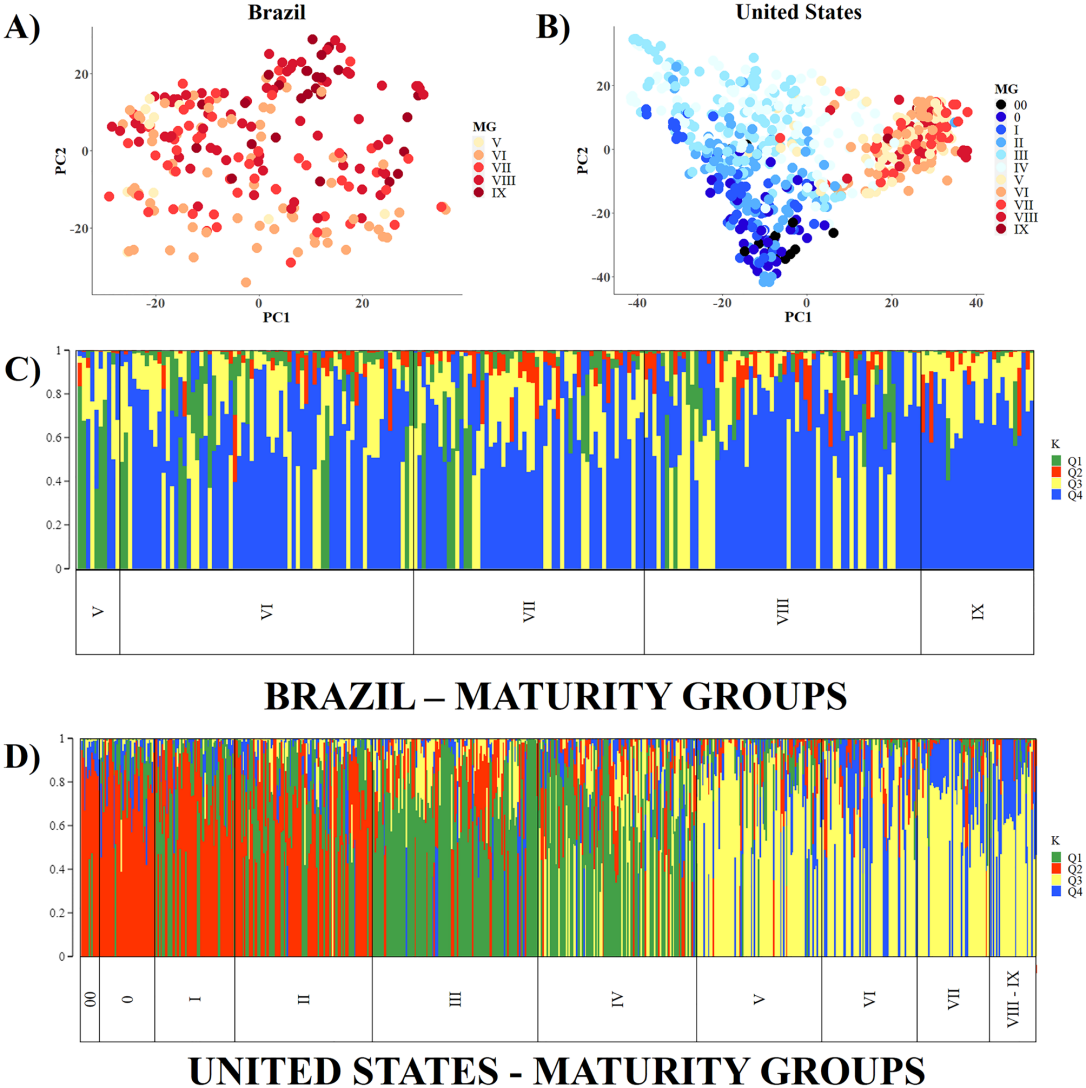
Population structure analysis of Brazilian and US cultivars according to their maturity groups. Principal component analysis (PCA) within Brazilian (**A**) and US (**B**) germplasms for each maturity groups; population structure of the Brazilian (**C**) and the US (**D**) genetic basis arranged according to their maturity groups.

In general, the Brazilian germplasm showed few differences between maturity groups (Supplementary Table S1 and Fig. 2A). This was also observed when we generated a population structure analysis exclusively with these cultivars (Fig. 2C). In contrast, the US germplasm showed a high variation of genetic distance when we analyzed their maturity groups (Supplementary Table S1) with a clear clustering of cultivars (Fig. 2B), which is more obvious when we observed their exclusive population structure analysis (Fig. 2D). The results show that early cultivars tend to be genetically distant from late cultivars in the US. The maturity groups from the southern-breeding program of the US (V, VI, VII, VIII, and IX) tend to be less genetically divergent versus northern groups (00, 0, I, II, III, and IV). This agrees with previous studies indicating distinct Northern and Southern genetic pools in the US^6^. There is a low divergence among US soybean cultivars from maturity groups higher than V (Fig. 2B). In contrast, cultivars from MG 00 and 0 were more genetically distant from cultivars of MG III and IV while maturity groups I-II were an intermediate group. The population structure analysis showed a high influence of Q2 in cultivars with MG 00-II. For cultivars in MG III and IV, we observed an increase of Q1. Finally, there is a high influence of Q3 in cultivars with maturity groups higher than V, which agrees with the genetic distance data.

### Meaningful genetic change of the Brazilian soybean germplasm occurred in modern genetic materials

The results demonstrate that both genetic bases had few increases in genetic distance among modern genetic materials (releases after 2000) when compared to cultivars from the 1950s to 1970s (Supplementary Table S2). According to the IBS genetic distance mean, the Brazilian genetic base was more diverse over the decades compared to US germplasm especially when we compared cultivars released before the 1970s and released after the 2000s (Supplementary Table S2).

**Figure 3:**
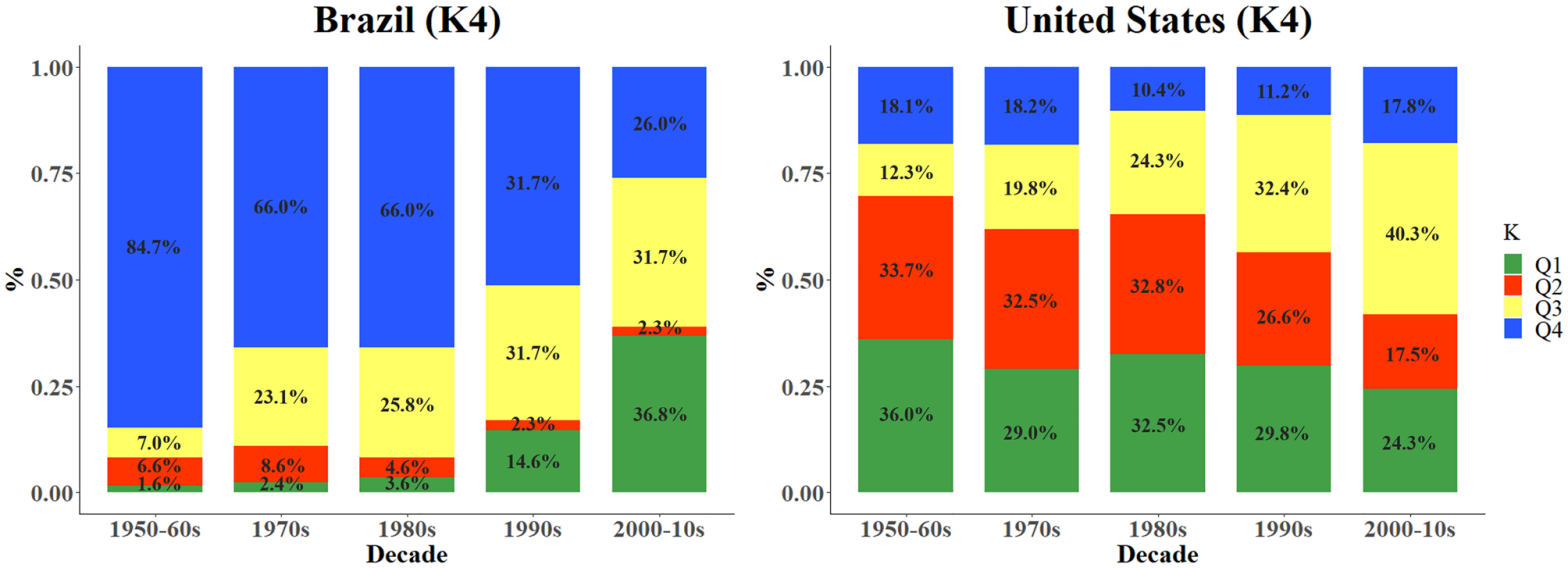
Mean assignment coefficients of the Brazilian and US cultivars belonging to the different decades of release (1950 to 2010) to STRUCTURE groups (Q1, Q2, Q3, and Q4) considering K = 4.

Average assignment coefficients (Q1, Q2, Q3, and Q4) from genetic structure results were calculated for both germplasm pools. All accessions were sorted according to their origin and decade of release (Fig. 3). We observed high genomic modifications over the decades in the Brazilian germplasm. Modern genetic materials (2000–2010) had Q1, Q2, Q3, and Q4 values of 36.8%, 2.3%, 31.7%, and 26.0%, respectively, while old accessions (1950-1960s) had means of Q1, Q2, Q3, and Q4 equal to 1.6%, 6.6%, 7.0%, and 84.7%, respectively. A high decrease was observed for Q4 starting in the 1990s whereas Q1 and Q3 highly increased during the same period. For the US genetic base, we observed an increase of Q3 and a decrease of Q2 over time. Old cultivars (1950–1970) had Q1, Q2, Q3, and Q4 values of 36.0%, 33.7%, 12.3%, and 18.1%, respectively, while modern cultivars (2000–2010) had Q1, Q2, Q3, and Q4 of 24.3%, 17.5%, 40.3%, and 17.8%, respectively.

**Figure 4:**
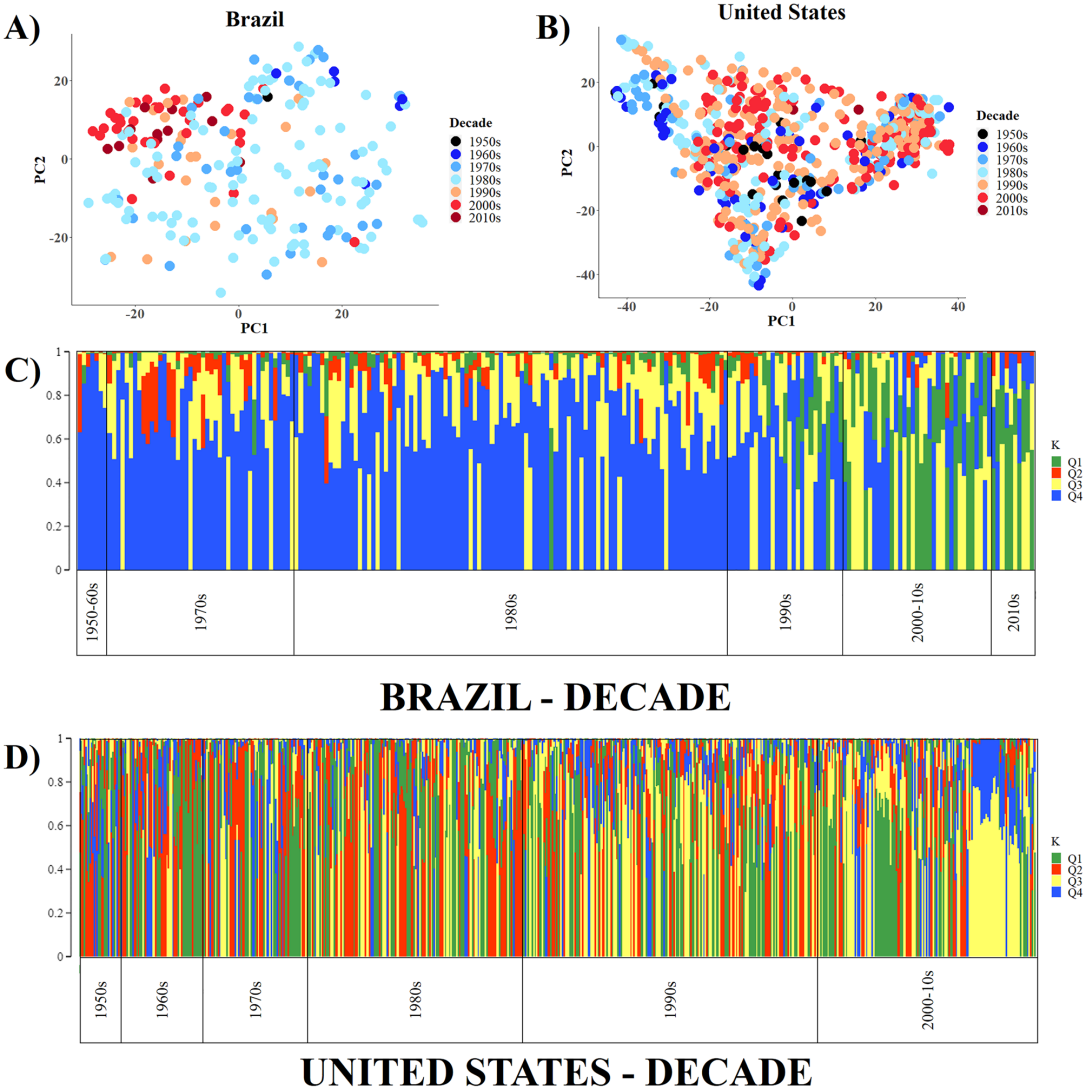
Population structure of Brazilian and US cultivars according to their decade of release. Principal component analysis (PCA) within Brazilian (**A**) and US (**B**) germplasm for each decade; population structure of the Brazilian (**C**) and the US (**D**) genetic bases arranged according to their decade of release.

Modification during the 1990s became more evident upon analysis of the PCA and genetic structure results of the Brazilian genetic base considering the decades of release (Fig. 4A and C). We observed an increase in the influence of the Q2 in modern genetic materials (2000–2010) when we compared the results to old genetic materials (1950–1970). In contrast, the US genetic base showed few variations over time according to the average of genetic distance (Supplementary Table S2), PCA, and the exclusive population structure analysis (Fig. 4B and D). These results suggest a large influence of new alleles in the Brazilian germplasm after the 1990s.

### Maturity genes under selection between Brazilian and US cultivars

Seventy-two SNPs with F_ST_ ≥ 0.4 between Brazilian and US cultivars were identified (Supplementary Table S3). These SNPs are located on chromosomes 1, 4, 6, 7, 9, 10, 12, 16, 18, and 19 (Supplementary Figure S2). Twenty-six 100-Kbp genomic regions with a high degree of diversification between Brazilian and US genetic bases were also found (Table 1). The results for Tajima’s D showed that these regions had balancing events that maintained the diversity of their bases. Two regions on chromosome 6 (47.3 – 47.4 Mbp and 47.3—47.4 Mbp) and another on chromosome 16 (31.10—31.20 Mbp) had few variations in Brazilian accessions (Supplementary Table S4). In contrast, the allele distribution for most of the SNPs present in these genomic regions in US germplasm was higher compared to Brazilian germplasm. An opposite scenario was observed for the other three regions located on chromosomes 7 (6.30 – 6.40 Mbp), 16 (30.70 – 30.80), and 19 (3.00 – 3.10) (Supplementary Table S4). The allele variance was higher in the Brazilian genetic base than US germplasm for these three intervals.

**Table 1:** Summary of the genomic regions with high F_ST_ values between Brazilian and US germplasms.

Chr.^a^	Start (Mbp)^b^	End (Mbp)^c^	SNP^d^	F_ST_		Tajima’s D^g^			π (10E^−05^)^h^		
				(High)^e^	(Reg.)^f^	ALL	BR	US	BR	US	US/BR^i^
1	48.70	48.80	5	0.45	0.47	4.07	2.45	2.47	1.76	1.60	0.91
4	50.20	50.30	7	0.44	0.19	4.12	2.12	3.93	1.89	2.86	1.51
6	0.60	0.70	6	0.40	0.32	4.20	1.42	3.53	1.58	2.38	1.50
6	46.90	47.00	8	0.41	0.29	4.24	1.70	3.55	2.18	2.92	1.34
6	47.30	47.40	4	0.40	0.42	4.19	− 0.03	3.83	0.58	1.80	3.10
6	47.40	47.50	9	0.41	0.39	5.58	0.37	5.08	1.73	4.08	2.36
6	47.50	47.60	4	0.49	0.35	3.35	1.16	2.84	0.81	1.47	1.82
6	47.70	47.80	16	0.46	0.22	5.42	2.30	5.23	3.85	6.81	1.77
6	47.80	47.90	15	0.40	0.29	5.87	2.98	4.97	5.05	6.20	1.23
6	48.10	48.20	20	0.44	0.17	5.82	2.64	5.61	6.10	8.63	1.42
6	48.40	48.50	4	0.47	0.15	1.94	1.15	1.72	0.80	1.08	1.35
7	6.30	6.40	6	0.44	0.16	1.32	2.34	0.78	1.63	0.90	0.55
9	41.50	41.60	7	0.40	0.17	4.34	1.82	4.55	1.52	3.13	2.06
10	44.20	44.30	6	0.52	0.23	2.95	2.61	2.00	2.13	1.63	0.77
10	44.40	44.50	7	0.44	0.16	3.84	3.05	2.90	2.66	2.58	0.97
12	6.10	6.20	9	0.46	0.10	4.99	3.92	5.22	3.83	3.83	1.00
16	3.00	3.10	12	0.42	0.09	1.74	2.25	1.24	3.27	2.26	0.69
16	29.40	29.50	10	0.45	0.12	4.63	3.96	4.24	3.86	4.01	1.04
16	30.70	30.80	6	0.41	0.30	2.21	2.96	0.97	2.30	1.03	0.45
16	31.10	31.20	6	0.51	0.27	3.38	0.55	3.18	0.98	2.20	2.24
18	48.60	48.70	5	0.42	0.32	2.76	4.00	1.20	2.45	1.12	0.46
18	57.20	57.30	9	0.46	0.17	2.76	3.42	2.03	3.21	2.13	0.66
19	0.90	1.00	7	0.40	0.11	2.65	3.40	2.12	2.15	1.97	0.92
19	3.00	3.10	5	0.42	0.39	2.21	4.08	0.34	2.45	0.76	0.31
19	3.10	3.20	4	0.40	0.42	2.84	3.23	1.25	1.78	0.94	0.53
19	3.40	3.50	4	0.40	0.42	2.84	3.23	1.25	2.24	1.31	0.58

^a^Soybean chromosome.

^b^Start position of the genomic region with high F_ST_ values.

^c^End position of the genomic region with high F_ST_ values.

^d^total number of SNPs observed in this interval.

^e^The highest F_ST_ value observed in a SNP of this interval.

^f^The genomic region average F_ST_.

^g^Tajima’s D coefficient for all (ALL), Brazilian (BR), and United States (US) germplasms.

^h^Nucleotide diversity values for all (ALL), Brazilian (BR), and United States (US) germplasms.

^i^nucleotide diversity ratio between the populations.

**Figure 5:**
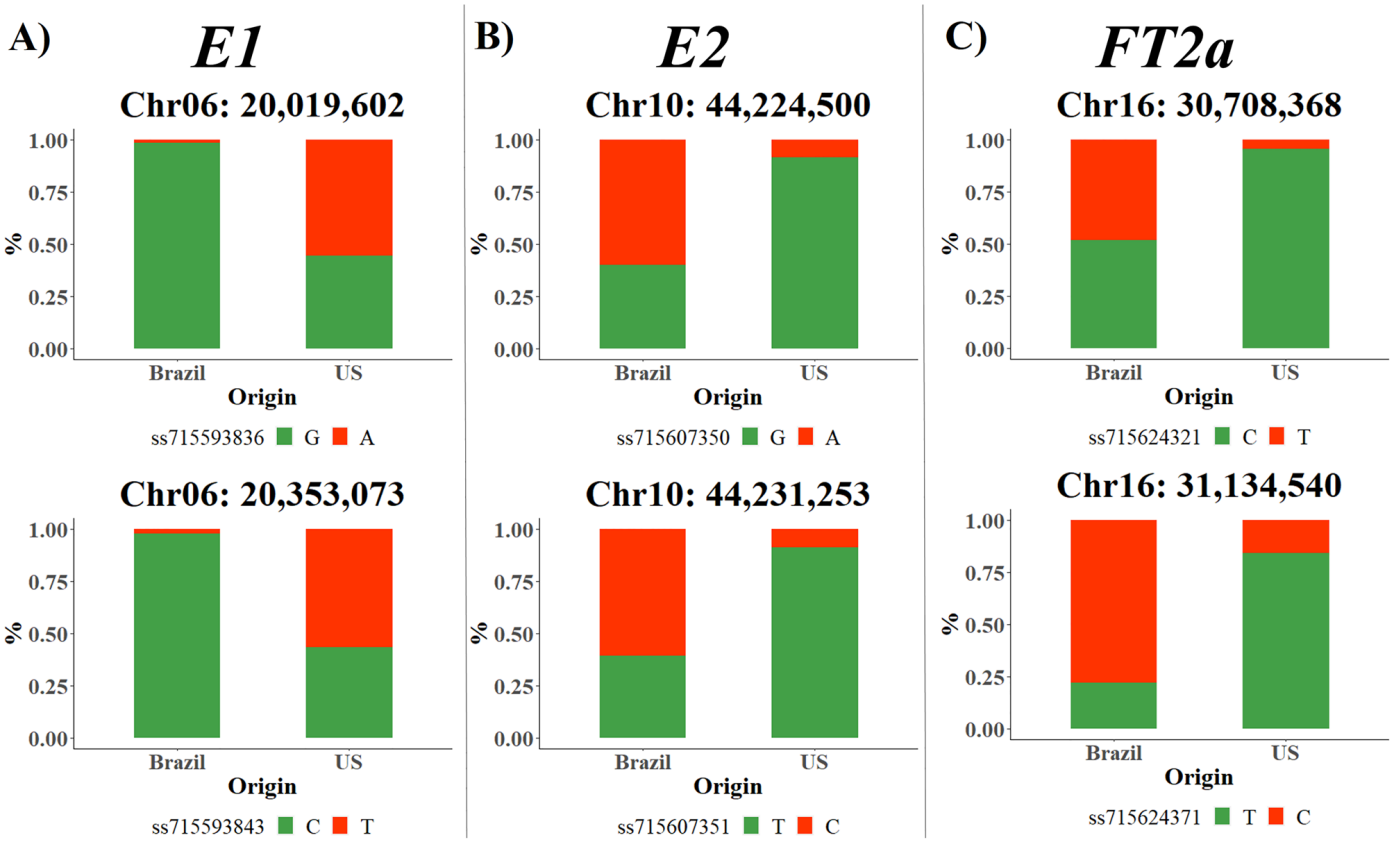
The allele frequency distribution for SNPs close to loci (**A**) *E1* (chromosome 6), (**B**) *E2* (chromosome 10), and (**C**) *FT2a* (chromosome 16) in Brazilian and US germplasms.

Six SNPs located close to maturity loci *E1* (Chr06: 20,207,077 to 20,207,940 bp)^14^, *E2* (Chr10: 45,294,735 to 45,316,121 bp)^15^, and *FT2a* (Chr16: 31,109,999 to 31,114,963)^16^ had a large influence on the differentiation of the Brazilian and US genetic bases (Fig. 5). For the SNPs ss715607350 (Chr10: 44,224,500), ss715607351 (Chr10: 44,231,253), and ss715624321 (Chr16: 30,708,368), we found that the alternative allele was barely present in US germplasm whereas the Brazilian genetic base had an equal distribution between reference and alternative alleles. When we examined the SNPs ss715624371 (Chr16: 31,134,540) and ss715624379 (Chr16: 31,181,902), the frequency of the alternative allele remains low in the US germplasm. However, the alternative alleles of these two SNPs were present in more than 78% of the Brazilian accessions in contrast to the previous three SNPs. Finally, the alternative allele for SNPs ss715593836 (Chr06: 20,019,602) and ss715593843 (Chr06: 20,353,073) were extremely rare in Brazilian germplasm with only 2% of the accessions carrying them. In contrast, the US germplasm had an equal distribution of reference and alternative alleles in their accessions. However, all accessions with the alternative alleles belonged to MGs lower than VI with less than five cultivars in MG V.

Ten SNPs were identified related to the gene’s modifier mutations present in Brazilian and US germplasm; these were distributed on chromosomes 4, 6, 10, 12, 16, and 19 (Supplementary Table S5). These SNPs had differing allele frequencies and could distinguish both genetic bases. Six modifications had a clear influence on the maturity of the accessions whereas two of these had a large influence in some decades of breeding (Supplementary Figure S3). The SNP ss715593833 had a similar haplotype as two SNPs described as close to the *E1* loci (ss715593836 and ss715593843) due to the linkage disequilibrium (LD) among them. At the end of this chromosome, we also observed another three relevant SNPs in LD: ss715594746, ss715594787, and ss715594990. In the US germplasm, we observed a decrease in the alternative allele in accessions with MG values lower than IV. We detected other relevant modifications on chromosome 12 for SNPs ss715613204 and ss715613207. Both SNPs had a minor allele frequency higher than 0.35 in Brazilian germplasm with an increase in the alternative allele in cultivars with MGs higher than VII. In contrast, alternative alleles for both SNPs were extremely rare in the US germplasm except for accessions with MG higher than VII.

There were 312 genomic regions that differentiate northern (00 – IV MG) and southern (V – IX MG) cultivar groups (Supplementary Table S6), which included the *Dt1* locus. We compared the SNPs observed in the genomic region close to the *Dt1* gene (Chr19: 45.20—45.30 Mbp) with the growth habit phenotype data available for 284 lines at the USDA website (www.ars-grin.gov). The phenotypic data suggests that these SNPs are associated with growth habit. Moreover, our diversity analysis demonstrated a putative selective sweep for the *Dt1* gene in the northern germplasm, which has the dominant loci fixed for *Dt1*; the southern lines tend to be more diverse compared to the northern US cultivars (Supplementary Table S7). In contrast, other genomic regions have lower nucleotide diversity in southern accessions compared to the northern accessions. An important disease resistance gene cluster was observed on chromosome 13 bearing four loci: *Rsv1*, *Rpv1*, *Rpg1*, and *Rps3*^17–20^. In this interval, we observed two genomic regions (29.70 – 29.80 Mbp and 31.90 – 32.00 Mbp) under putative selective sweeps in the southern germplasm (Supplementary Table S8).

Besides these regions, 1,401 SNPs with F_ST_ values higher than 0.40 between northern and southern US cultivars were also identified (Supplementary Table S9). In addition, there were 23 SNPs with F_ST_ values higher than 0.70 spread on chromosomes 1, 3, 6, and 19. Seven of them were located close to another important soybean locus: *E1* (involved in soybean maturity control) (Supplementary Table S10). These SNPs clearly differentiate northern and southern US cultivars with the reference allele fixed in northern genetic materials, and the alternative alleles in southern accessions. Gene modification in US germplasm was also detected in our study. One hundred twenty-six SNPs were identified in F_ST_ analysis modifying 125 genes (Supplementary Table S11).

Finally, we detected 1,557 SNPs with F_ST_ values higher than 0.40 between super-early cultivars (00 – 0 MG) and early cultivars (III – IV MG) (Supplementary Table S12). Seventeen SNPs had F_ST_ values higher than 0.70 spread on chromosomes 4, 7, 8, and 10. The SNPs identified on chromosome 10 were close to the *E2* locus. We also detected 168 SNPs associated with modifications in 164 genes (Supplementary Table S13).

### Genetic diversity was higher in Brazilian modern cultivars than founder lines

We observed two SNPs with large differences in allelic frequencies in the Brazilian germplasm (Supplementary Figure S4). On chromosome 4, SNP ss715588874 (50,545,890 bp) had a decrease of the allele “A” in cultivars released after 2000 with only nine of the 45 Brazilian cultivars with this allele. A similar situation was observed on chromosome 19 for ss715633722 (3,180,152 bp) with half of the modern accessions having the presence of allele “C”. Both SNPs had similar distribution according to their decades in the US genetic base with a large influence of reference alleles.

There were 126 genomic regions spread on almost all soybean chromosomes in Brazilian cultivars. The only exception was chromosome 20 (Supplementary Table S14). Our analysis between cultivars released before and after 1996 identified 30 putative regions under breeding sweep events. Thirteen regions had a decrease in diversity in modern genetic cultivars according to Tajima’s D and π results. Two genomic regions observed were close to important disease resistance loci: one on chromosome 13 (30.30 – 30.40 Mbp) close to the resistance gene cluster (with *Rsv1*, *Rpv1*, *Rpg1*, and *Rps3*)^17–20^ and another on chromosome 14 (1.70 – 1.80 Mbp) with a southern stem canker resistance loci^21,22^. In contrast, thirty-one genomic regions had an increase in diversity in modern cultivars, which suggested putative introgression events in these accessions. Two genomic regions were observed, on chromosome 2 (40.90 – 40.10 Mbp) and 9 (40.30—40.40 Mbp). These were previously reported to have an association with ureide content and iron nutrient content, respectively^23,24^.

Besides these regions, there were also 409 SNPs with F_ST_ values higher than 0.40, distributed across all soybean chromosomes. There were 73 SNPs with F_ST_ values higher than 0.70 (Supplementary Table S15). Some of these SNPs were also reported to be associated with important soybean traits such as plant height, seed mass, water use efficiency, nutrient content, and ureide content^23–27^.

We also identified gene modifications with a high impact on the Brazilian genetic base when we compared cultivars according to their decade of release. Of the 409 SNPs identified in F_ST_ analysis, we observed 40 SNPs causing modifications in 39 soybean genes (Supplementary Table S16). Three SNPs with F_ST_ values higher than 0.70 were associated with non-synonymous modifications: ss715588896 (*Glyma.04G239600* – a snoaL-like polyketide cyclase), ss715607653 (*Glyma.10g051900* – a gene with a methyltransferase domain), and ss715632020 (*Glyma.18G256700* – a PQQ enzyme repeat).

## Discussion

Soybeans were domesticated in China from its annual wild ancestor [*Glycine soja* (Sieb. and Zucc.)] more than 5,000 years ago^28^. US soybean history began in colonial times as a forage crop, but breeding programs began in the early 1900s. During the 1940s and 1950s, US soybean-breeding programs grew in importance and aimed to change plant architecture, maturity, seed quality, and yield. Most of the cultivated soybean came from the public sector until the early 1980s when private companies became an important and leading source of soybean cultivars in the US^29–31^.

The US soybean breeding history is longer than the Brazilian breeding history. The first report of soybeans in Brazil was from 1882 in the state of Bahia, but the first released cultivars were from the 1950s in states of São Paulo and Rio Grande do Sul. Brazilian public and private institutes were responsible for most of the cultivars released in Brazil until the 1990s. As soybean production in Brazil became more relevant—along with a more favorable scenario of intellectual property rights—multinational companies began expanding their soybean breeding programs in the country^32^.

Here, we compared Brazilian and US germplasm over decades and identified four genetic groups in the population structure analysis. When we compared Brazilian population structure, we found that the Q1 genetic group had a large influence in modern genetic materials. Q1 was evenly distributed in the US germplasm over decades. These results might indicate that similar alleles from US germplasm were incorporated into modern Brazilian cultivars. Furthermore, modern cultivars from both germplasms had similar assignments for Q1, Q3, and Q4, which might represent allele introgressions into Brazilian germplasm though soybean-breeding programs. The emergence of new companies brought new lines from other germplasm pools, which might explain the meaningful change in the modern Brazilian cultivars compared to those released before 1990^32^.

In contrast, the US genetic base did not show large modifications over decades according to the population structure results. However, when we analyzed the US germplasm according to their maturity groups, it was possible to identify three clusters among the cultivars. The first group was represented by early cultivars (MG = 00, 0, I, and II) with a large influence of Q2 in this germplasm pool; Q3 and Q4 were barely present. The second group was formed by cultivars with MG III, and IV with Q1 having a large influence on the US soybean germplasm. The third group was comprised of cultivars with MG higher than V: This group had a large influence of Q3 in the germplasm. These results indicate that maturity genes largely influence the US genetic base. Similar results were observed in another study that analyzed 579 soybeans from the US and Canada. These were clustered into the same three groups that we identified^33^. Our analysis showed an increase of 230 cultivars from other panels, but there was no modification in the genetic structure of the US germplasm even with the addition of new samples.

The comparison between the Brazilian and US genetic bases identified 72 SNPs with high F_ST_ values in 11 chromosomes. Some of these SNPs were located on three known maturity loci: *E1*, *E2* and *FT2a*, which have a large impact on soybean maturity. The *E1* locus was previously cloned and identified as a transcription factor with a region distantly related to B3 domain (*Glyma.06g207800*)^14^. A map-based cloning strategy was used to show that the *E2* locus was homologous to the cloned Arabidopsis GIGANTEA protein (Glyma.10g221500)^15^. *FT2a* (Glyma.16g150700), previously described as *E9* locus, has been associated with flowering control and soybean adaptation to different photoperiodic environments in other studies^16,34^. Previous studies proposed that *E1* acts as a repressor and has an important role in controlling photoperiodic expression patterns of *FT2a* loci^35,36^. *E2* recessive alleles could not suppress the *FT2a* loci expression, which directly impacts soybean flowering with early plants^15^.

Wolfgang et al. identified that the *E1* recessive allele was predominant in northern germplasms, and along with the *E2* recessive allele were not present in southern germplasms (MG higher than V)^31^. US founder lines with MG lower than I had a unique influence of *E2* locus on their background compared to the founder lines with MG values higher than III ^33^. In Canada, soybean cultivars were concentrated on MGs lower than II. The *e2* recessive allele was under selection in Guelph cultivars and fixed in Ridgetown accessions^37^. Large F_ST_ values were also observed when Chinese germplasms were compared to the US and Canadian genetic bases^10^. Our results corroborate previous studies and suggest that these three loci play different roles in Brazil and US germplasm. One explanation for this finding might be associated with the large number of US cultivars with MG values lower than V. This increases the need for genes conditioning early maturity. Brazilian accessions only belonged to MG higher than V, which decreases the need for cultivars with recessive maturity *E* loci for adaptation in most parts of the country. This scenario is different from the US, which has a large soybean area using cultivars with MG lower than V. However, SNPs close to FT2a locus were extremely rare in the US germplasm. These data demonstrate that maturity loci have different roles in both germplasms.

The analysis between Brazilian and US germplasm also revealed eight SNPs with high F_ST_ values. Five of them were previously associated with four important soybean traits: yield, maturity, water-use efficiency, and shoot-nutrient concentration^23,25–27,38–40^. Interestingly, four of these SNPs were practically fixed in US germplasms, except for ss715593829 (shoot-potassium content and water-use efficiency), which has an equal distribution of alleles. On the contrary, the Brazilian genetic base fixed the “T” allele (reference allele) for ss715593829 but has an equal allele distribution for ss715588874 (seed weight), ss715613207 (seed weight and yield), and ss715624268 (maturity). Finally, we found that the alternative allele for SNP ss715624371, which is related to maturity, was fixed in Brazilian accessions. Thus, the genotypic differences detected among the SNPs with high F_ST_ values observed here might represent the geographical and adaptive modifications present in Brazilian and US soybean germplasms.

The US germplasm concentrated its diversity into differences among maturity loci. Our results demonstrate that *E1* has a major role in differentiating northern (00 – IV) and southern (V – IX) germplasms. Similar results were observed in a previous study ^31^. We further observed that the *E2* locus has a large impact in differentiating early and super-early cultivars similar to prior studies^31,33,37^. Other important loci that differentiate the US germplasm were observed in our results, such as the *Dt1* locus that appears to have fixed the dominant allele in northern cultivars. Our results represent breeding efforts to improve soybean cultivars to most US regions.

Historically, the Brazilian soybean accessions have gone through several modifications. Concerning morphological traits, modern Brazilian soybeans tend to be earlier, more productive, shorter, with a lower number of branches per plant, and lower lodging score than old cultivars^41^. Moreover, modern Brazilian cultivars remove more nutrients from the soil versus older accessions (except for calcium and sulfur). There was a meaningful impact for magnesium and nitrogen in grain nutrient concentration within a 10-year perspective. High-yielding Brazilian modern cultivars could remove more potassium (21.4%) and less nitrogen (4.3%) versus older varieties^42^. We identified 126 genomic regions that differentiate older and modern cultivars. Similar results for regions on chromosomes 7, 17, and 18 were described previously in the Brazilian germplasm^8^. We also identified 409 SNPs with F_ST_ values higher than 0.40 versus cultivars released before 1996 and after 1996. There were 14 SNPs previously reported in other studies that were related to maturity, seed mass, water-use efficiency, plant height, ureide content, and shoot-nutrient content (Supplementary Table S15)^23–27^. Four SNPs (ss715582676, ss715582689, ss715603946, and ss715603949), were putative introgressed genomic regions in modern genetic materials. They were associated with ureide and shoot-iron content. These results are associated with other studies and indicated that modern genetic materials incorporated nutrient absorption alleles associated with new architecture, maturity, and yield genes. In turn, these features impact modern Brazilian cultivar diversity.

**Figure 6:**
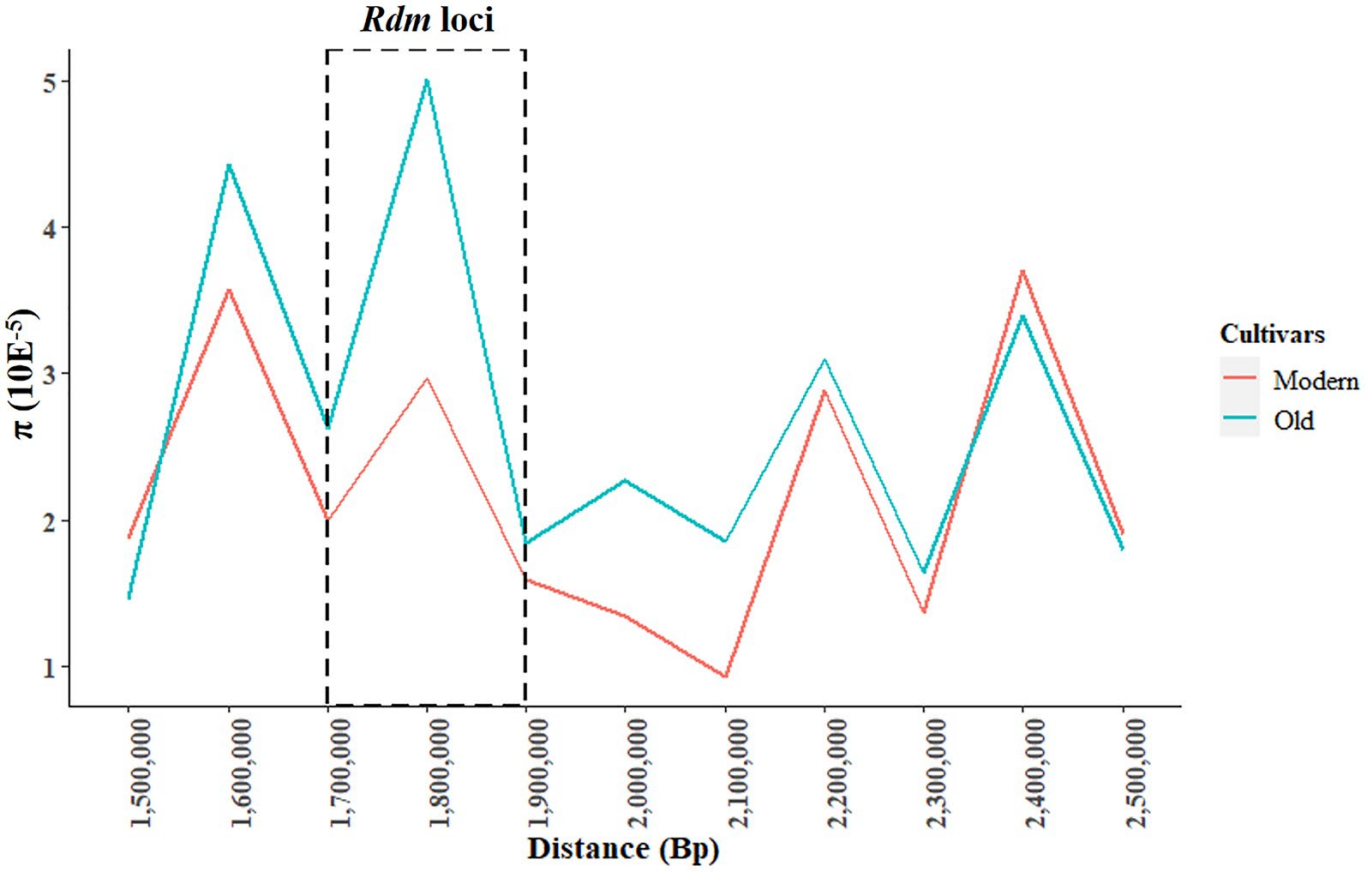
Nucleotide diversity (π) between modern and old cultivars of the southern stem canker resistance loci (*Rdm*).

Southern stem canker was an historically important soybean disease responsible for losses of 1.8 million metric tons in Brazil in 1994 alone^43^. A massive introgression of resistance genes to control this pathogen was necessary. We found some phenotypic results from 43 Brazilian accessions used in another study. Most of the genetic materials released after 1996 were reported to be resistant to *Diaporthe aspalathi* while there was phenotypic variation among old cultivars. We analyzed the mapping region associated with southern stem canker resistance^22^ and observed eight SNPs with F_ST_ values of 0.56, which had a perfect correlation between phenotypic and genomic data (Supplementary Table S17). Moreover, ss715617869 (Chr14:1,731,256) and ss715617951 (Chr14:1,938,019) were also associated with southern stem canker in another study^21^. Our results showed that this region underwent a strong decrease in diversity in modern genetic materials versus old genetic materials (Fig. 6). This suggests a selective sweep region that breeders incorporated into modern Brazilian seed lines.

In summary, we identified factors that differentiate germplasm from Brazil and the US. Maturity loci play a more important role in the US germplasm compared to Brazil due to the large number of MGs in the US. There is a clear influence of major *E* loci on the MGs of the US germplasm. In contrast, the Brazilian genetic base appears to have more influence from the incorporation of new lines from others germplasm pools^32^. The population structure analysis suggests a major change in Brazilian germplasms after 1996. Moreover, our results suggest that the US germplasm appears to be more diverse than the Brazilian germplasm, even with a narrow base, as described in other studies^44–46^. Both germplasm pools could benefit from increases in useful genetic diversity, especially modern Brazilian cultivars due their narrow genetic base. The F_ST_ demonstrates that some regions are related to adaptation, maturity and productivity traits that might have been influenced by this change. We also observed important genomic regions that were under selection such as the southern stem canker locus that demonstrate the importance of breeding programs to solve the impact of pathogens on crop productivity. Our study generated more information regarding the soybean adaptation to the world’s two major soybean producers. Finally, these results offer new insights into the genomic regions that should be the focus of breeding programs to adapt new lines and generate competitive cultivars.

## Methods

### Soybean genetic data

This study used 230 Brazilian cultivars and 675 US cultivars from different maturity groups and time periods (Supplementary Table S18). These cultivars were previously genotyped with the SoySNP50K panel^47^. We also extracted public information from other cultivars^8,48–50^. The entire dataset was obtained from the Soybase website^50^. To obtain a consensus genomic information, we only selected SNPs in SoySNP50K. The SNPs used in this study were referenced to version 2 of the soybean genome (Glyma.Wm82.a2 – Gmax2.0)^11^, and only biallelic variation was maintained in the final panel. SNPs with minor allele frequency (MAF) and call rates (CR) lower than 0.05, and 0.8, respectively, were removed.

### Population structure analysis

In the original panel, we removed SNPs with linkage disequilibrium higher than 0.30 via plink 1.09 software with the “–indep-pairwise” option^51^. This step removed the allele variation with linkage disequilibrium and used 1,798 SNPs for analysis. The structure software^52^ was used to generate the analysis with a 100,000 burn-in period, and 100,000 Markov Chain Monte Carlo (MCMC) repetitions for K from 1 to 10. Ten runs were performed for each analyzed K, and we used Structure Harvester to define the two best delta K values based on the Evanno criterion^53^. We used STRUCTURE PLOT software to generated all the structure bar plots^54^. The same SNPs were used for principal component analysis (PCA) between Brazilian and US genetic bases using TASSEL 5.0 software^55^.

### Distance matrix analysis between Brazilian and US genetic bases

To compare the genetic divergence in Brazilian and US germplasms, we created an identity-by-state (IBS) genetic distance matrix using TASSEL 5.0 software^55^ We removed alleles with a minor allele frequency (MAF) lower than 0.05. We separated the cultivars according to their geographic origin, maturity groups, and decade of release.

### Genetic diversity analysis

We grouped the cultivars according to their location, maturity groups, and release date. We used vcftools software for each analysis^56^. We used the population fixation index coefficient (F_ST_), nucleotide diversity coefficient (π), and the Tajima’s D coefficient to detect genomic regions under selection^57,58^. We performed three analyses: a) Brazilian accessions vs US accessions; b) among Brazilian cultivars; and c) among US cultivars. For each analysis, we generated the F_ST_ per SNP, and 100-kbp sliding window size for π, Tajima’s D, and F_ST_.

### Genetic annotation of the genomic regions under selection

We used SnpEff and SnpSift programs to identify the possible allelic variation observed for each SNP identified in diversity studies^59^. The SnpEff software was used for annotation of the vcf file. We used the SnpSift program with the perl script vcfEffOnePerLine.pl to generate a matrix with one effect per line. We only considered SNP modifications that were influenced directly in genes such as start and stop codons, splice site, and exons.

## Supplementary Information

Supplementary Information 1.

Supplementary Information 2.
