# Supplementary material for: Genetic relationships and genome selection signatures between soybean cultivars from Brazil and United States after decades of breeding
Source: Sci Rep. 2022 Jun 23;12:10663. doi: 10.1038/s41598-022-15022-y (PMC9226155; doi:10.1038/s41598-022-15022-y)
Supplement: Supplementary file 1 — Supplementary Information 1. [file 41598_2022_15022_MOESM1_ESM.pdf]

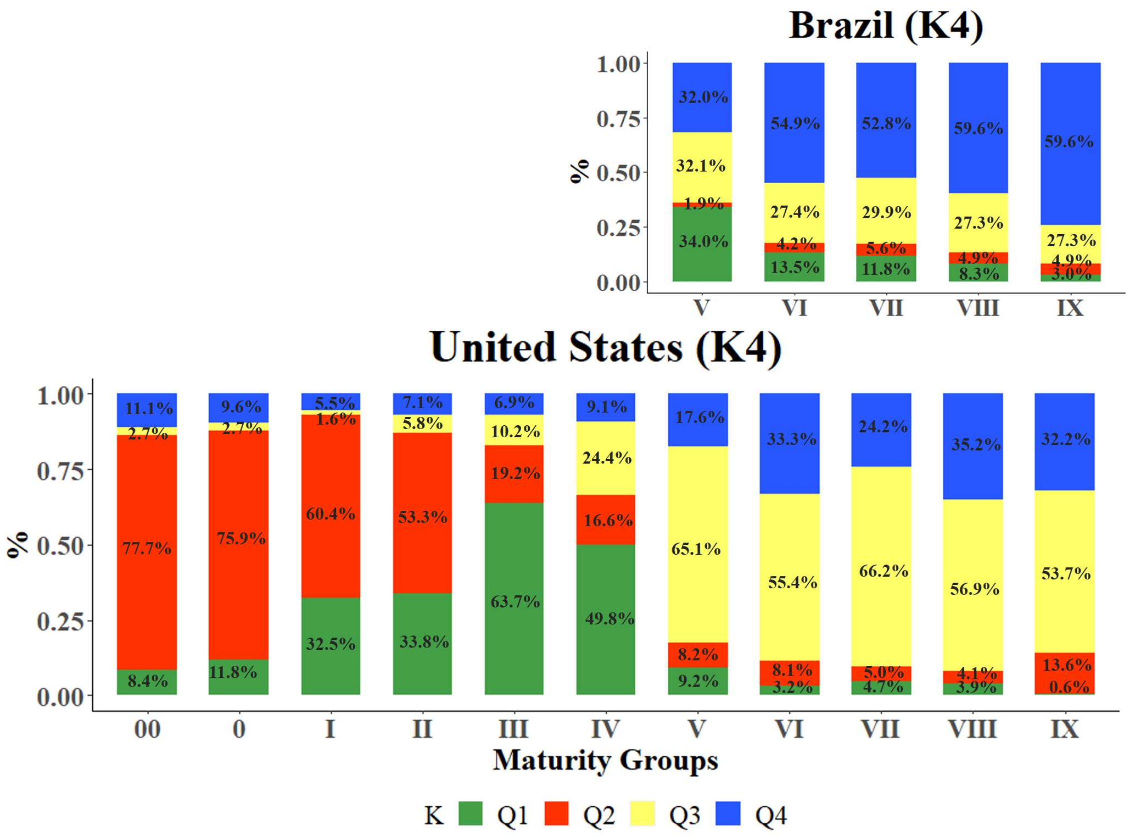

Supplementary Figure S1: Mean assignment coefficients of the Brazilian and US cultivars belonging to the different maturity groups (00 to IX) and STRUCTURE groups (Q1, Q2, Q3, and Q4) considering K = 4.

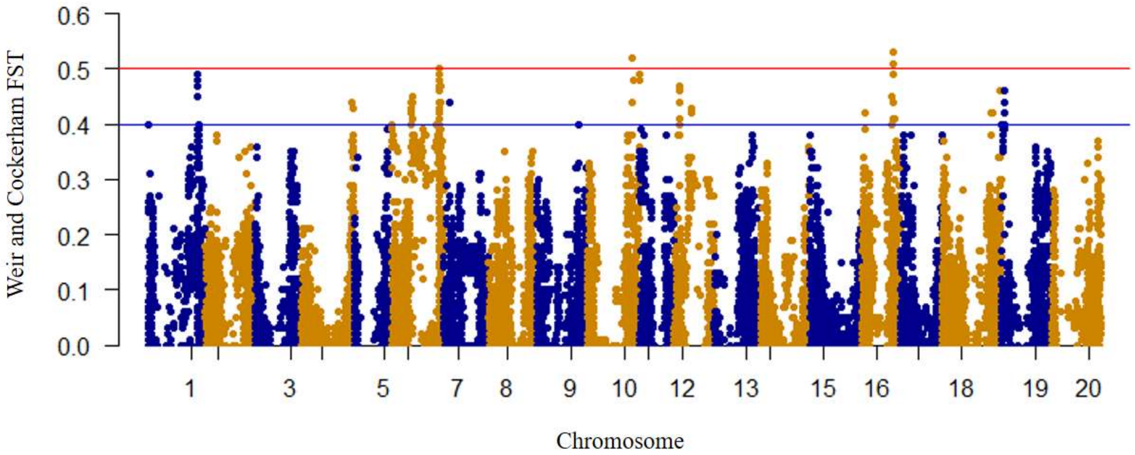

Supplementary Figure S2: Weir and Cockerham F_{ST} between Brazilian and US cultivars of 21,800 SNPs over 20 soybean chromosomes.

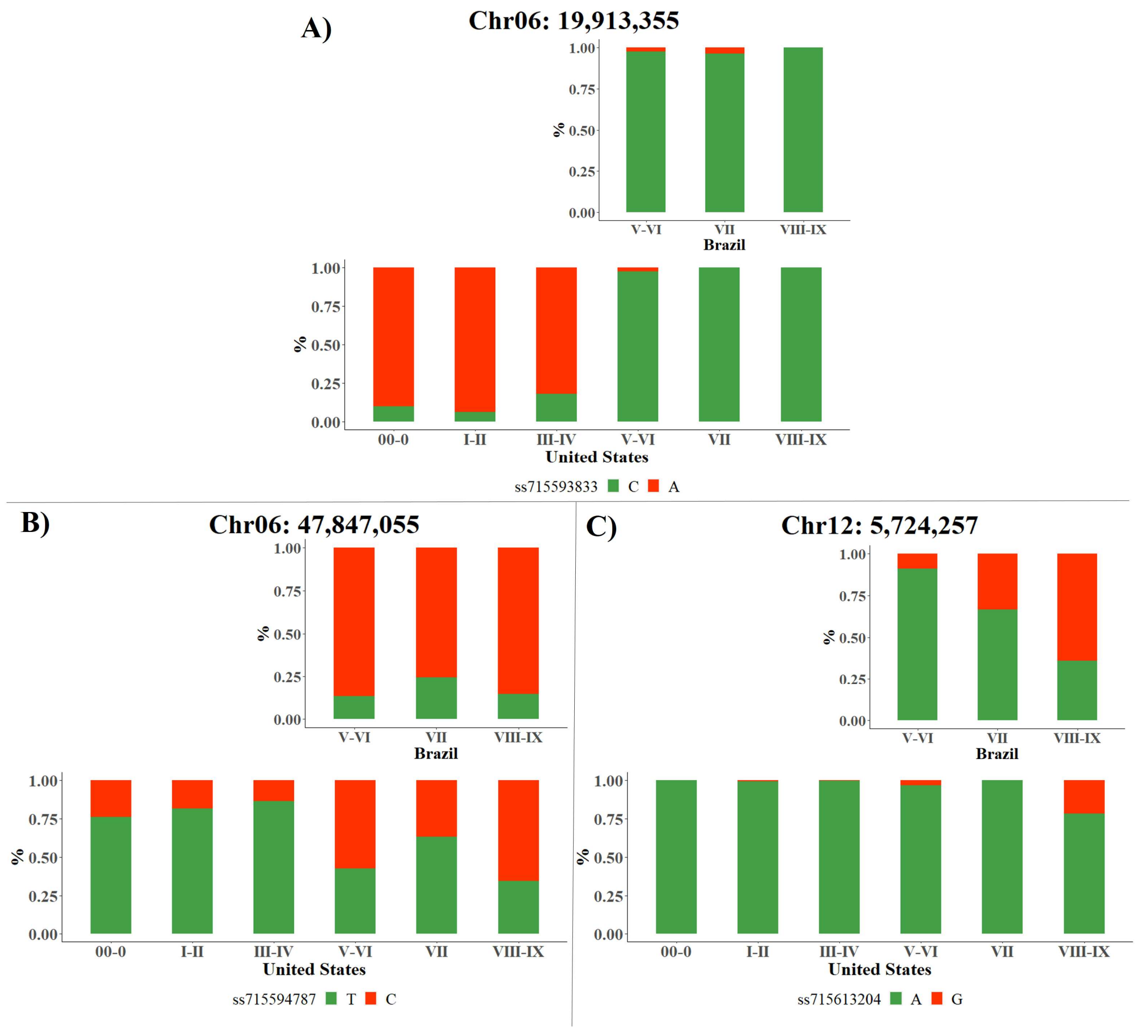

Supplementary Figure S3: The allele frequency distribution of (**A**) ss715593833 (chromosome 6), (**B**) ss715594787 (chromosome 6), and (**C**) ss715613204 (chromosome 12) in Brazilian and US germplasm according to their maturity groups.

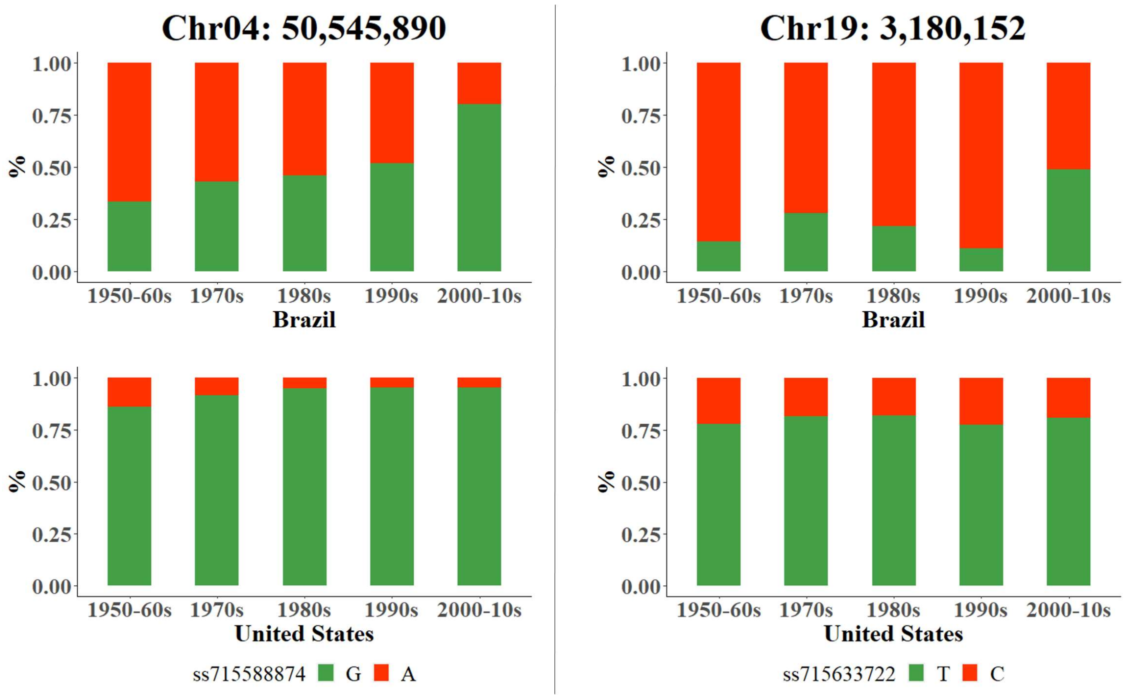

Supplementary Figure S4: The allele frequency distribution of (**A**) SNP ss715588874 (chromosome 4) and (**B**) SNP ss715633722 (chromosome 19) in Brazilian and US germplasm according to their release decade.
